# Oral findings and craniofacial morphology in a patient with Schwartz‐Jampel syndrome and severe obstructive sleep apnea: A case report

**DOI:** 10.1002/ccr3.3193

**Published:** 2020-08-09

**Authors:** Mevadee Pibulniyom, Patimaporn Pungchanchaikul, Thanate Assawakawintip, Supakit Peanchitlertkajorn

**Affiliations:** ^1^ Srinakharinwirot University Panyananthaphikkhu Chonprathan Medical Center Nonthaburi Thailand; ^2^ Faculty of Dentistry Khon Kaen University Khon Kaen Thailand; ^3^ Department of Orthodontics Faculty of Dentistry Mahidol University Bangkok Thailand

**Keywords:** bimaxillary hypoplasia, case report, pediatric obstructive sleep apnea, posterior crossbite, Schwartz‐Jampel syndrome

## Abstract

We reported dental and craniofacial characteristics of an SJS patient with severe OSA. Not only does the syndrome cause skeletal abnormalities and myotonia, but it also affects the craniofacial development resulting in a severe constriction of maxillary arch and bimaxillary retrognathia which may increase a risk to develop pediatric OSA.

## Graphical abstract

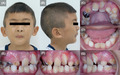

## 1 INTRODUCTION

This is a report of an 8‐year‐old male with Schwartz‐Jampel syndrome with severe obstructive sleep apnea. Dental findings include severe maxillary crowding, posterior crossbite, missing lower incisors, and supernumerary lower premolar. Significant craniofacial characteristics include Type I skeletal relationship with bimaxillary hypoplasia, hyperdivergent skeletal pattern, and severe constriction of dental arches.

Schwartz‐Jampel Syndrome Type 1 (SJS1, ORPHA:800; OMIM#255800) is a very rare genetic disorder with prevalence less than 1:1 000 000.^1^ SJS, named after Schwartz and Jampel (1962)^2^, is a genetic disorder caused by mutations in *HSPG2* gene. This gene encodes for perlecan, a cellular matrix protein regulating muscle contraction and chondrogenesis.^3^ A defective *HSPG2* (OMIM*142 461) gene results in prominent SJS features of prolonged muscle contraction (myotonia) and multiple skeletal abnormalities (chondrodysplasia). Facial dysmorphology is distinctive and rather consistent across ethnicities.^4^ There are approximately 130 SJS reports in the current literature with only a few describing dental findings. Díaz‐Serrano et al.^5^ delineated oral manifestations such as dental crowding, high‐arched palate, multiple impacted teeth and TMJ problem found in two siblings with SJS. Mallineni et al.^6^ reported a Chinese boy with SJS who presented with bulbous crowns with marked cervical constriction, amber‐brown discoloration, brittle enamel, soft dentine, and severe attrition in his primary teeth. A macrodontic primary incisor and a congenitally missing permanent incisor were also described in the same patient.^6^ In this paper, we present an 8‐year‐old male patient with SJS and severe obstructive sleep apnea (OSA). To the best of our knowledge, this is second SJS case with OSA reported in the literature after Cook and Borkowski.^7^ In addition to oral findings, a craniofacial morphology of this patient will be presented.

## 2 CASE PRESENTATION

An 8‐year‐9‐month‐old male patient of normal intelligence with SJS was referred to the Department of Orthodontics, Faculty of Dentistry, Mahidol University, Thailand. He is a child of a healthy nonconsanguineous Thai couple. His birthweight was 3.070 kg with unremarkable prenatal history. Generalized muscle stiffness and abnormal gait have been observed since his first year. The diagnosis based on SJS’s cardinal signs was made at 3 years of age. His medical history includes hypothyroidism, repeated hospitalization due to severe allergy and respiratory distress, and severe OSA. He regularly receives carbamazepine to relieve his muscle contraction, and levothyroxine for hypothyroidism. Parents noticed occasional snoring at 6 months old and became worsened with increasing age. Other nocturnal symptoms included enuresis, choking, and gasping for air. Daytime symptoms include frequent morning drowsiness and headache, nausea, hypersomnolence, as well as poor concentration. He was referred to a sleep physician for a diagnostic polysomnography at the age of seven. The diagnosis for severe OSA was confirmed with an apnea‐hypopnea index of 29 events/hour. The continuous positive airway pressure (CPAP) was prescribed to relieve his OSA symptoms prior a referral for a dental appliance.

**Figure 1:**
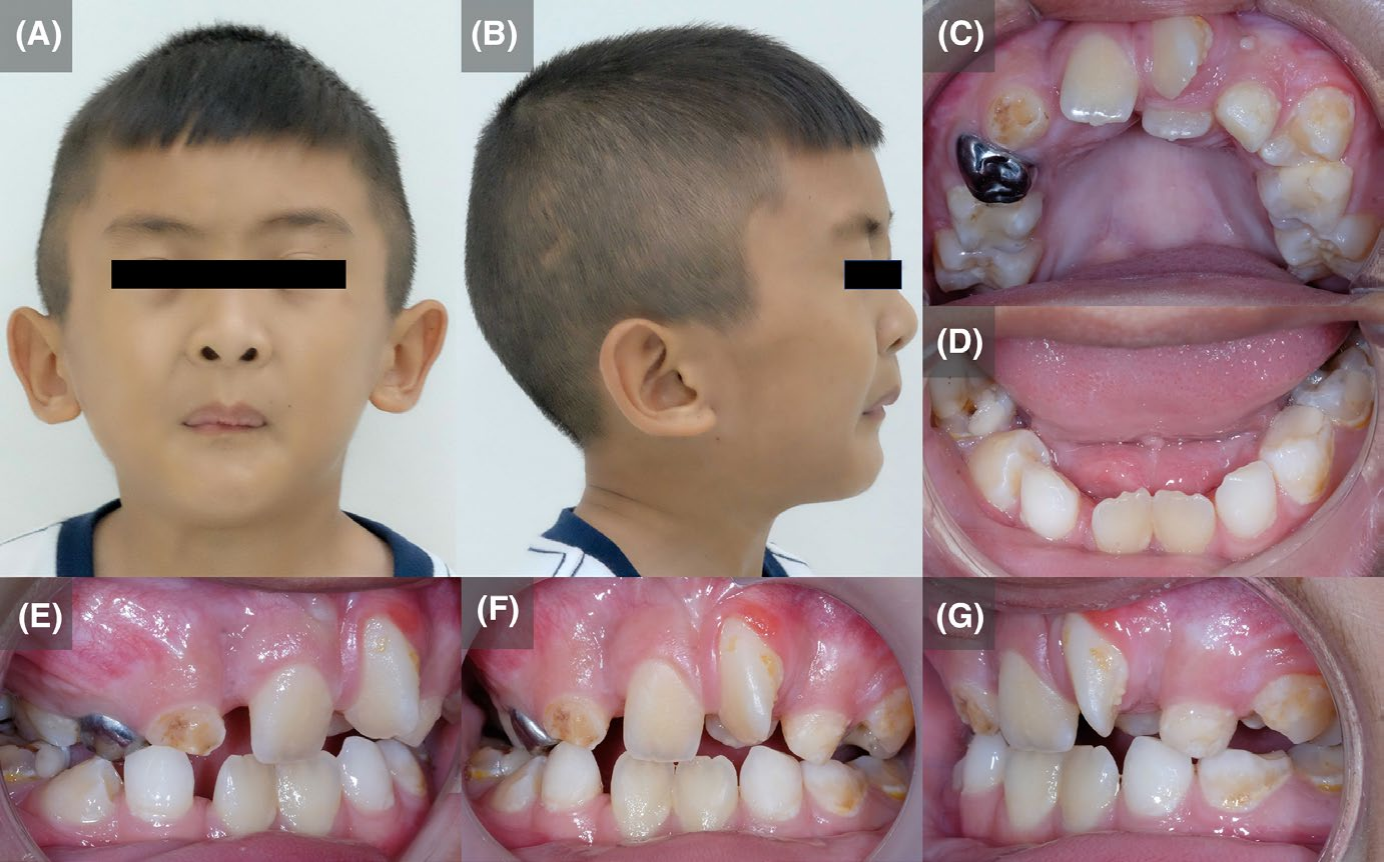
Extraoral and intraoral pictures. Facial frontal (A), Facial profile (B), Maxillary occlusal (C), Mandibular occlusal (D), Right occlusion (E), Frontal occlusion (F), and Left occlusion (G)

**Figure 2:**
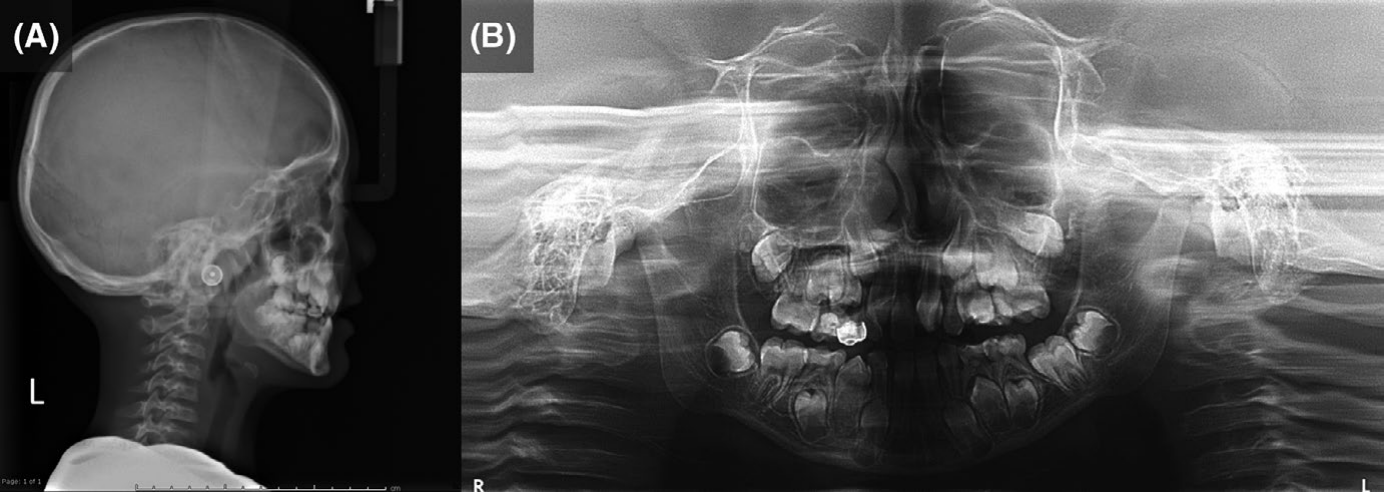
Cephalometric (A) and Panoramic radiographs (B) of the patient

On physical examination, the patient had a proportionate short stature with his weight in the tenth percentile. He presented with multiple skeletal deformities, and generalized muscle stiffness. Extraoral examination revealed a distinctive mask‐like facie, blepharophimosis, low‐set ears, microstomia and pursed lip, limited mouth opening (25 mm), asymmetrical oval facial form, convex facial profile and bimaxillary retrognathia (Figure 1). On oral examination, the patient was in mixed dentition with very severe dental crowding especially in the upper arch. The amount of dental crowding was measured at 12 and 6 mm for the upper and lower arch respectively. There was untreated active caries noted. The maxillary arch was severely constricted with a very high‐vaulted palate. The upper intermolar width was significantly reduced with a measurement of 36 mm. Bilateral posterior crossbite was observed (Figure 1). Dental development was within normal range. Permanent mandibular lateral incisors were clinically missing. Enlarged palatine tonsils were present in the retropharyngeal area. Radiographic examination confirmed congenital hypodontia of the lower laterals and supernumerary mandibular teeth at the premolar region bilaterally (Figure 2). Cephalometric analysis demonstrated that the patient had Type I skeleton relationship with retrognathic maxilla and mandible. The mandible rotated posteriorly while the maxilla exhibited counter‐clockwise rotation. This creates a hyperdivergent pattern of the jaw bones. Both upper and lower incisors are retroclined and retruded. Soft tissue measurements showed the upper and lower lip were in a retrusive position following the underlying skeletons. The detailed cephalometric characteristics of the patient are presented in Table 1.

**Table 1:** Cephalometric measurements of the patient with SJS

	Cephalometric measurements	Norm	SD	Pre‐ treatment	Interpretation
Skeletal	Antero‐posterior:				
	SNA	84	3.58	78.5	Retrognathic maxilla
	SNB	81	3.59	77	Retrognathic mandible
	ANB	3	2.50	1.5	Skeletal type I
	A to N perpend	4.59	3.86	‐5	Retrognathic maxilla
	Pg to N perpend	0.47	5.96	‐1.1	Orthognathic mandible
	Vertical:				
	NS‐MP	30	5.61	39	Posterior rotation of mandibular plane
	NS‐PP	9	3.03	‐1	Anterior rotation of palatal plane
	MP‐PP	21	5.25	40	Open configuration
Dental	Antero‐posterior:				
	U1‐NA	22	5.94	6.5	Retroclined U1
	U1‐NA (mm)	5	2.13	‐1	Retruded position of U1
	L1‐NB	30	5.61	9	Retroclined L1
	L1‐NB (mm)	7	2.22	0	Retruded position of L1
Soft tissue	H‐angle	14	3.83	5	Retruded position of upper lip
	Lower lip to E‐plane	2	2.03	‐2	Retruded position of lower lip

## 3 DISCUSSION

Three types of SJS:1A,1B, and 2 have been reported with distinct severity of skeletal malformation and clinical course.^8^ Mutations of *HSPG2* caused types 1A and 1B. The latter is more severe and recognizable at birth. Type 2 SJS, currently termed as Stüve‐Wiedemann Syndrome, caused by mutation of *LIFR* gene, encoding for a transmembrane protein. The phenotype of SJS2 could be apparent at birth with short‐limb dysplasia and bowing of long bone. Definite diagnosis should be confirmed by family genotyping. However, an early childhood onset, facial dysmorphology; blepharospasm, narrow palpebral fissures, small mouth with pursed lips, permanent myotonia, and short stature of this patient matched the Type 1A phenotype described in previous reports. Although SJS has been mostly suggested to be inherited by autosomal recessive, the dominant pattern is confirmed in a few families.^9^, ^10^, ^11^ The mutation underlying these phenotypes could be a result of dominant negative effect of perlecan.^12^

Dental crowding is usually a result of the discrepancy between tooth and jaw size. In this case report, the severe dental crowding was likely a result of constricted jaw bones rather than larger teeth since the patient exhibited very narrow maxillary and mandibular arches. This also demonstrates that SJS could have effects not only on general bones but also craniofacial skeletons. Good oral hygiene helps prevent premature loss of primary teeth that could worsen the arch length discrepancy. Therefore, routine dental care and regular dental check‐up should be emphasized. The patient's maxilla is constricted anteriorly and even more so laterally. The mandible is also retrognathic. This could illustrate the effects of myotonia on craniofacial development. Because maxillary and mandibular hypoplasia could be a risk factor in developing pediatric OSA.^13^ This could mean that patients with SJS are at risks to develop OSA as the patient in this report. However, this correlation has not been previously raised, possibly due to the rarity of SJS. This is the first case report of SJS from Thailand. It is also the first SJS report with a detailed description of craniofacial morphology.

## 4 CONCLUSION

SJS is a rare genetic syndrome with only a few reports of oral findings in the literature. The patient presented with typical features of SJS includes myotonia, short structure, abnormal gait, mask‐like facie, blepharophimosis, microstomia, retrognathia, and pursed lip. In addition, he was also diagnosed with a severe Obstructive Sleep Apnea which affected his daily activities and quality of life. We reported his oral findings that included severe dental crowding and narrow maxillary and mandibular arches. This is also the first study describing detailed craniofacial characteristics of a patient with SJS. The clinical findings of this patient suggest that SJS could have effects not only on general but also on craniofacial skeletons. And myotonia of craniofacial muscles could results in a diminished maxillomandibular development. Since maxillary and mandibular hypoplasia is a risk factor for pediatric OSA, patients with SJS could be at risk to develop OSA as the patient in this report.

## CONFLICT OF INTEREST

None declared.

## AUTHOR CONTRIBUTIONS

SP: conceived the ideas and supervised the treatment. TA, and SP: collected the data SP, MP, and PP: analyzed the data SP, MP, and PP: led the writing.
